# Contacts for Molybdenum Disulfide: Interface Chemistry and Thermal Stability

**DOI:** 10.3390/ma13030693

**Published:** 2020-02-04

**Authors:** Keren M. Freedy, Stephen J. McDonnell

**Affiliations:** Department of Materials Science and Engineering, University of Virginia, Charlottesville, VA 22904, USA; kmfreedy@gmail.com

**Keywords:** transition metal dichalcogenides, semiconductors, nanoelectronics, contacts, interface chemistry, contact resistance, thermal boundary conductance, X-ray photoelectron spectroscopy

## Abstract

In this review on contacts with MoS_2_, we consider reports on both interface chemistry and device characteristics. We show that there is considerable disagreement between reported properties, at least some of which may be explained by variability in the properties of geological MoS_2_. Furthermore, we highlight that while early experiments using photoemission to study the interface behavior of metal-MoS_2_ showed a lack of Fermi-level pinning, device measurements repeatedly confirm that the interface is indeed pinned. Here we suggest that a parallel conduction mechanism enabled by metallic defects in the MoS_2_ materials may explain both results. We note that processing conditions during metal depositions on MoS_2_ can play a critical role in the interface chemistry, with differences between high vacuum and ultra-high vacuum being particularly important for low work function metals. This can be used to engineer the interfaces by using thin metal-oxide interlayers to protect the MoS_2_ from reactions with the metals. We also report on the changes in the interfaces that can occur at high temperature which include enhanced reactions between Ti or Cr and MoS_2_, diffusion of Ag into MoS_2_, and delamination of Fe. What is clear is that there is a dearth of experimental work that investigates both the interface chemistry and device properties in parallel.

## 1. Introduction

The last 15 years have seen a renewed interest in van der Waals solids with a new focus on their potential in nanoelectronic applications. These materials have a long history of use as dry lubricants [1] and have been previously studied for their photoelectrochemical [2,3,4] and photovoltaic [5] properties. While there have been prior reports on monolayer 2D materials including ‘a single carbon hexagonal layer’ [6] and ‘single-layer MoS_2_’ [7], it was the seminal work of Novoselov and Geim [8,9] that triggered this remarkable interest in monolayer 2D for nanoelectronics. Since the isolation of monolayer graphene and the demonstration of its electronic properties [8,9,10], the interest in 2D materials beyond graphene has also been increasing. These 2D materials beyond graphene include hexagonal boron nitride (hBN), transition metal dichalcogenides, Silicene/Germanene/Stanene, as well as group III and group IV metal chalcogenides such as GaSe or SnS_2_ [11,12,13]. Similarly, despite monolayer MoS_2_ being exfoliated as early as 1986 [7], it was the demonstration of a monolayer MoS_2_ based transistor [14] that sparked an exponential rise in publications on the properties, synthesis, and electronic device applications of MoS_2_ [15]. A fundamental component of any electronic device is the metal contact that controls the flow of current and heat to external circuitry. This review article will cover the interface chemistry and properties of metal contacts to semiconducting 2D materials, with a primary focus on the metal-MoS_2_ interface. The role of processing conditions will also be discussed. Table 1 captures a summary of some of the metal-MoS_2_ research that has been carried out. It becomes clear that while there are many interface chemistry studies as well as studies focused on device properties, there is a lack of correlation studies that concurrently investigate chemistry, device properties and effects of processing.

## 2. Transition Metal Dichalcogenides

**Figure 1:**
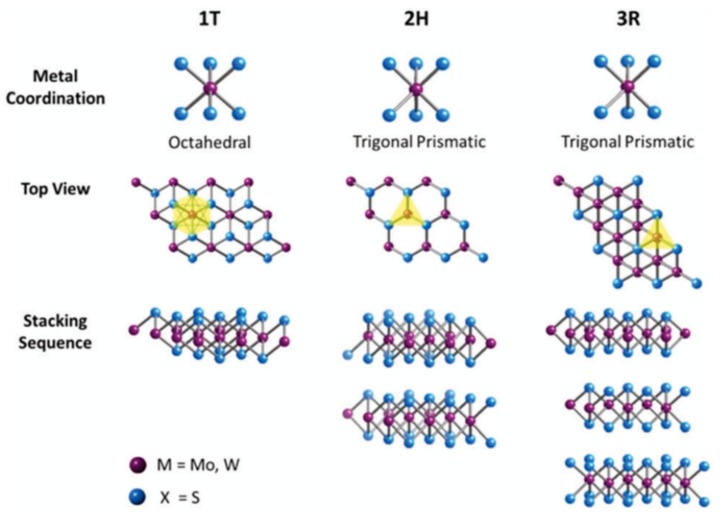
Metal coordination and stacking sequences of transition metal dichalcogenide (TMDC) structural unit cells. Metal coordination can be either octahedral or trigonal prismatic. The octahedral coordination allows stacking sequences which yield a tetragonal symmetry (1T). Dissimilar stacking sequences of trigonal prismatic single layers can give rise to different symmetries: hexagonal symmetry (2H) and rhombohedral symmetry (3R). Reproduced from R.J. Toh et al. Chem Commun., 2017, 53, 3054 – Published by The Royal Society of Chemistry.

MoS_2_ is the most commonly studied member of the transition metal dichalcogenide (TMDC) family of layered materials. Layered materials are defined as solid materials that are held together in part by secondary bonding such as van der Waals forces. By data mining the Materials Project Database, more than 1000 ‘weakly bonded’ materials have been identified [39]. These included both layered materials and also one-dimensional chains. The TMDC family takes the form of MX_2_ where M is a transition metal and X is a chalcogen (S, Se, or Te). This is illustrated in Figure 1. Unlike graphene, which is a flat layer of carbon with all covalent bonds existing on a 2D plane, a single ‘layer’ of a TMDC is actually three atomic layers thick and consist of an X-M-X. This layer is then held to other layers via van der Waals forces. In the case of MoS_2_, there exist three polytypes that are shown in Figure 1. The 1T and also the distorted 1T’ phase are metallic and of particular interest for catalysis [40,41,42] and also low resistance contacts [43]. The semiconducting 3R polytype can be generated through process control and has recently be shown to have comparable performance to 1T with respect to hydrogen evolution reactions [44]. However, it is the 2H phase that is thermodynamically stable and is therefore the most common polytype studied.

The TMDC family of materials exhibits a range of electronic properties including semiconductivity, semimetallic behavior, and superconductivity. They have a long history and many aspects have been covered in other reviews. A detailed review of their structure and properties was carried out in 1969 [1]. More recent reviews include those focused on combinations of synthesis, applications, and functionalization [15,45,46,47,48,49,50,51,52,53]. This review will focus specifically on the metal–TMDC interface chemistry and thermal stability.

## 3. Contacts for Nanoelectronics

Much of the recent focus on the metal–TMDC interfaces has been largely motivated by the goal of achieving Ohmic or low resistance contacts for electronic devices. A conventional approach to low resistance contacts stems from the Schottky Mott model, which predicts that the height of the barrier for electron injection is dependent on the degree of band bending at the metal/semiconductor interface [23,54]. This is quantified by the absolute value of the difference between the work function of the metal and the electron affinity of the semiconductor [55]. For an n-type semiconductor, the condition for an Ohmic contact is that the work function of the metal align in the conduction band of the semiconductor [56]. This condition results in no barrier to electron flow into the semiconductor. In practice Ohmic contacts are often achieved by satisfying the condition that the work function of the metal be less than that of the semiconductor to ensure a small barrier, and then complementing this by highly doping the contact area so that any barrier is sufficiently thin to allow easy tunneling. In fact, this approach was adapted and demonstrated for 2D materials by Chuang et al. [57]. The authors doped their TMDC layers (WSe_2_, MoS_2_, and MoSe_2_) with ~0.5% Nb. NbSe_2_ and NbS_2_ are metallic and so the effect was similar to degenerately doping the region under the contact. Based on the Schottky Mott model, metal contacts should be chosen based on work function to meet the condition for Ohmic contact. Given the propensity of MoS_2_ for n-type doping [58], the ideal candidate based on this model would therefore be low work function metal. The converse is true for WSe_2_ which is more likely to exhibit p-type doping [59] making high work function metals preferable for forming Ohmic contacts. Low work function metals including Ti (4.3 eV) [20,60], In (4.1 eV) [61], Mo (4.5 eV) [34], Cr (4.5 eV) [60], and Sc (3.5 eV) [20] may seem to be favorable candidates for MoS_2_ [48,61] High work function metals include Ni (5.0 eV) [20], Pt (5.9–6.1 eV) [20,62], Au (5.4–5.7 eV) [20,62], Pd (5.6 eV) [61]. 

It has been experimentally observed that metal–MoS_2_ interfaces rarely adhere to the behavior predicted by the Schottky Mott model [19,20,60,62,63] Contact behavior (Ohmic vs. Schottky or n-type vs. p-type) is found to be not entirely dependent on the work function difference between the semiconductor and the metal. For example, in the first report of a MoS_2_-based transistor, Radisavljevic et al. [64] report Ohmic behavior for Au contacts to n-type MoS_2_. Given the high work function of Au, this result is surprising. Similarly, Das et al. [20] investigated Sc, Ti, Ni and Pt contacts on MoS_2_ and showed that, despite markedly different metal work functions, all appear to be Fermi-level pinned to just below the conduction band. While, Kaushik et al. [25] observe the same n-type behavior for devices contacted with Au and Pd, Fontana et al. [65] show that Pd can form a p-type contact in agreement with Schottky Mott model, whereas Au forms an n-type contact in agreement with the results of others. 

**Figure 2:**
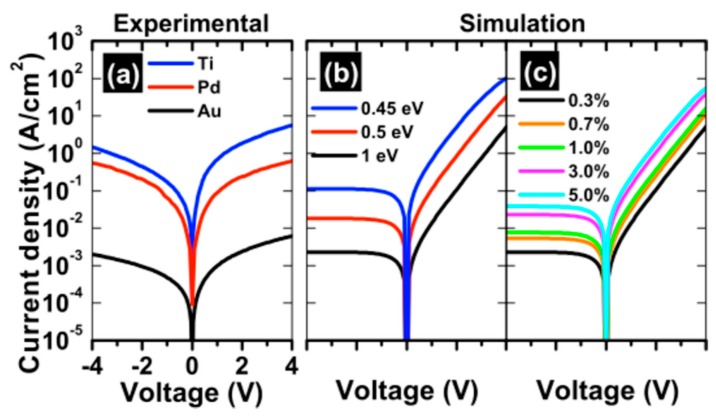
Comparison of the experimental and simulated IV characteristics. (**a**) Experimental current-voltage characteristics of Ti-MoS_2_, Pd-MoS_2_, and Au-MoS_2_ for comparison to the simulated curves (**b**,**c**). (**b**) Simulated IV characteristics for an inhomogeneous interface assuming fixed defect areal density of 0.3% with metal electron Schottky barriers of 0.45, 0.5, and 1 eV. (**c**) Fixed metal electron Schottky barrier of 1 eV and varying defect areal density of 0.3, 0.7, 1, 3, and 5%. Both (**b**) and (**c**) assume the defect electron Schottky barrier to be 0.4 eV and series resistance of 25 Ω. Reprinted with permission from McDonnell et al. ACS Nano 2014, 8, 3, 2880–2888. Copyright 2014 American Chemical Society.

It is apparent that two types of discrepancies exist in the literature concerning the electronic properties of metal-TMDC contacts. The first, as stated previously, is the deviation of experimental results from the Schottky Mott model. The model assumes that the two materials maintain their intrinsic properties upon contact. Given the absence of dangling bonds on the surface of TMDCs, they were believed to be chemically inert exhibiting minimal interactions with a metal overlayer [28]. This is in contrast with conventional semiconductors, like Si or group III-V materials such GaAs, which have surface dangling bonds that result in the formation of defect-induced or metal-induced gap states that pin the Fermi level [65,66] Gong et al. [62] suggest that in metal-MoS_2_ contacts, dipoles formed at the interface modify the metal work function, and that the S-Mo bonding is weakened by the adsorbed metal leading to the formation of states in the band gap of MoS_2_. McDonnell et al. [23] show that the presence of defects in geological MoS_2_, specifically Mo-rich clusters, are a likely explanation for the effective lowering of the Schottky barrier height in MoS_2_. These defects provide parallel conduction paths for the electrons. One would be the direct path from metal to MoS_2_ and the other would be metal-defect-MoS_2_. If the defect offers a low Schottky barrier, then even small areal densities of defects (on the order of 1%) will dominate the current-voltage characteristics of a contact due to the exponential dependence on barrier height as shown in Figure 2. This can manifest in the measurement of low electron Schottky barrier contacts even with high work function metals such as Au or Pt. The authors showed that even 0.3% surface coverage of defects was sufficient to explain the experimental observation of anomalously high reverse bias currents. Furthermore, Figure 2c demonstrates that local variations in defect concentration could have a significant impact on device-to-device variability. Additionally, a number of low work function metals including Ti, Mn, Ir, Sc, and Cr have been found to react with TMDCs [16,18,24,27,29,67]. Reaction products can also result in the creation of states in the TMDC band gap which pin the Fermi level [67]. Ultimately, the deviation from the Schottky Mott model is the result of different chemical and electronic interactions that occur at the metal/TMDC interface.

The second discrepancy in contact behavior is that which is found between different reports in the literature studying the same metal-TMDC systems. For example McDonnell et al. reported that two Au-MoS_2_ contacts on the same MoS_2_ crystal separated by only millimeters exhibited different behavior, with one suggesting p-type and the other n-type MoS_2_ [23]. Another example already mentioned is the n-type conduction observed by Kaushik et al. and the p-type conduction reported by Fontana et al. for Pd-MoS_2_ contacts. Similarly, English et al. report that Ti contacts behave worse than Ni contacts whereas Das et al. report the opposite. We note that the key finding in the paper by English et al. is that Au contacts deposited in UHV (~10^−9^ Torr) exhibit contact resistance that is three times less than that of Au contacts deposited in HV (~10^−6^ Torr). This illustrates that two seemingly identical metal-TMDC systems can exhibit different electronic properties due to different processing conditions, highlighting the important role of processing in interface properties that are often discussed in the literature as solely material-dependent. Processing effects also explain deviations between theory and experiment. For example, Chaung et al. show that MoO_x_ contacts to p-type MoS_2_ and p-type WSe_2_ exhibit Schottky barriers [68]. McDonnell et al. note that this deviates from band alignment calculations which predict Ohmic behavior [68]. The disagreement is attributed to the deposition of MoO_x_ in HV, where the deposition results in a higher concentration of carbon in the film yielding a lower MoO_x_ work function.

## 4. Interface Chemistry

An important and potentially dominant factor in metal–semiconductor contacts is the interface chemistry. Allain et al. [63] defined two potential metal–2D interfaces in their work. In a conventional metal–semiconductor interface, there are primary bonds between the metal and the semiconductor. However, for 2D materials, it is often assumed that there will be a van der Waals gap at the contact interface due to the lack of dangling bonds. Allain et al. [63] considered that the van der Waals interface was only one type of interface and that the other would be one were primary bonds did exist. They used Ti as an example of a metal that would form bonds to 2D materials. Clearly there actually exists a spectrum of interfaces that exist between these two extremes [17,69].

A recent review by Domask et al. [70] focuses on thermodynamic predictions of transition metal–MoS_2_ interface reactions. The key prediction is that many metals will react with MoS_2_. This is important because metal reactions with MoS_2_ would form an interface chemistry distinctly different from van der Waals interface. This is quite consistent with early experimental reports from the 1980s. For example, Kamaratos and Papageorgopoulos investigated Fe and Ni particles on the MoS_2_ surface. They found that both formed islands on the surface at room temperature [22,31]. McGovern et al. [16] and later Lince et al. [28] would both report on a range of metals on MoS_2_, studied by photoemissions spectroscopy. McGovern considered a range of metals and reported their calculated heats of reaction (Δ*H*_R_) for these metals with M oS_2_. Their focus was on reactive metals, but they acknowledged prior evidence [71,72] that Δ*H*_R_ values as high as 0.5 eV/atom may still result in reactions. Therefore, they studied Cu, Ni, and In with Δ*H*_R_ values from 0 to 0.5 eV/atom and Ti, Al, and Mg with Δ*H*_R_ values from −2.22 to 0 eV/atom. Their results showed that Cu and In were not reactive while Ti and Mg were reactive, as predicted. However, they saw that Ni did show some reaction while Al did not. Their results regarding Ni will be discussed in comparison to more results later, but with respect to Al, the authors concluded that either photoemission wasn’t sufficiently sensitive to detect reactions or that there were large kinetic barriers preventing it. The results of the study suggest that calculations based on bulk thermodynamics are not entirely predictive of reactivity for metal–TMDC systems.

Lince et al. [28] focused on measuring the band bending induced by metal depositions on MoS_2_ surfaces, with a discussion of interface chemistry. They considered Ag, Al, Au, Co, Fe, In, Mn, Pd, Rh, Ti, and V. Of these, they only saw reactions with Mn. This is in contrast to the earlier work of McGovern which reported Ti reactions with MoS_2_. Lince et al. [28] drew attention to this fact and speculated that their own evaporation of Ti may have resulted in some Ti oxidation because their depositions were carried out at 3 × 10^−8^ Torr, while McGovern et al. [16] used 2 × 10^−9^ Torr. This may seem insignificant, however, McDonnell et al. [16] would later show large differences in Ti depositions carried out under ~10^−9^ Torr and ~10^−7^ Torr conditions, while Freedy et al. [73] would show that even 10^−6^ Torr to 10^−7^ Torr could yield large changes in the Ti chemistry.

**Figure 3:**
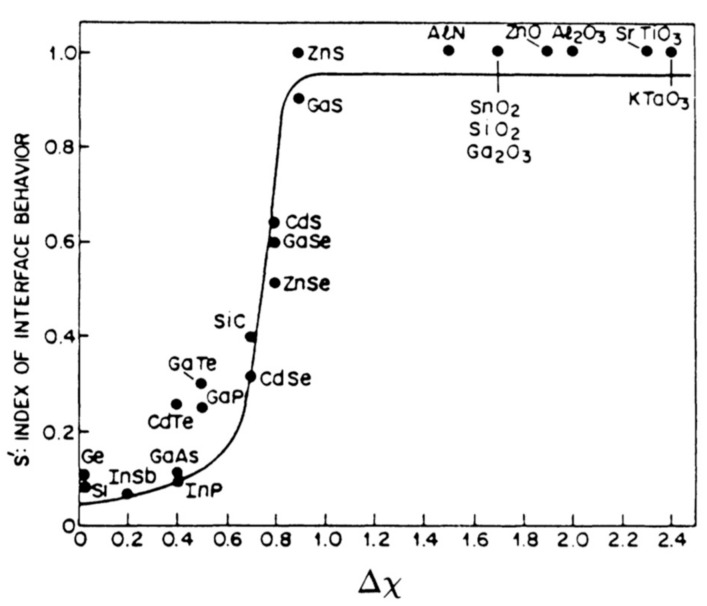
Collected data representing several independent experiments plotted as a function of lattice electronegativity difference Δχ. Reprinted with permission from Kurtin et al, Phys Rev Lett., 22, 1433 (1969). Copyright (1969) by the American Physical Society.

With respect to Fermi-level pinning, Lince et al. [28] used the MoS_2_ surface to test contemporary theories on Fermi-level pinning. In particular, the authors noted that prior work had shown that the ‘index of interface behavior’ which is defined as the Schottky barrier divided by the electron affinity of the metal contact S’ = (dφ_B_/dχ_M_), was shown to vary markedly between ionic and covalent materials [74]. For a metal–semiconductor system where the Schottky–Mott model is observed, the Schottky barrier would be linearly dependent on the metal work function (or electron affinity) and S’ should have a slope of 1. In cases of severe Fermi-level pinning, S’ would have a slope closer to 0.1. It had been shown in early work, that if one plots the S’ values obtained for a range of compound semiconductors against the electronegativity difference of the elements in the compound (which would indicate the degree of ionicity) there is a dramatic shift between S’ values close to 0.1 (high pinning) to S’ values close to 1 (little/no pinning) at Δχ values of ~0.7 eV as shown in Figure 3 [74]. This was deemed to be a transition between covalent and ionic character. In the work of Lince et al. [28]. the authors chose MoS_2_, with a Δχ value of only 0.42, to test whether or not the degree of Fermi-level pinning was related primarily to the bonding type of the semiconductor (ionic or covalent), or instead to the reactivity of the substrate. Interestingly the authors found that the index of interface behavior was S’ 1.28 ± 0.22 eV. The implications of this result, taken together with those of Das et al. [20] and McDonnell et al. [23] will be discussed later.

Durbin et al. [26] used soft x-ray photoemission to study the reactions between Cr and MoS_2_ during ultra-high-vacuum electron beam deposition and post deposition annealing. It was found that Cr reacted with the MoS_2_ to form metallic Mo metal, Cr with intermixed S and a sulfur rich surface [26,27]. The same group reported similar reactions with Mn, where MnS clearly formed in addition to metal Mo [29]. However, they also showed that, in contrast, Fe deposition resulted in only surface Fe-S phases and S-vacancy formation rather than bulk FeS formation [30]. They stated that these three elements followed the expectations of bulk thermodynamics, since the Fe reaction with MoS_2_ to form FeS or FeS_2_ yields a slightly positive ΔG of +3 and 14.1 kcal/mol, respectively, CrS would be −10.1 kcal/mol and MnS would be −25.2 kcal/mol, indicated that a reaction with Fe is not expected, while reactions with Cr and Mn should occur with Mn being stronger [26].

## 5. The Impact of Processing Conditions on Interface chemistry

### 5.1. Deposition Ambient

**Figure 4:**
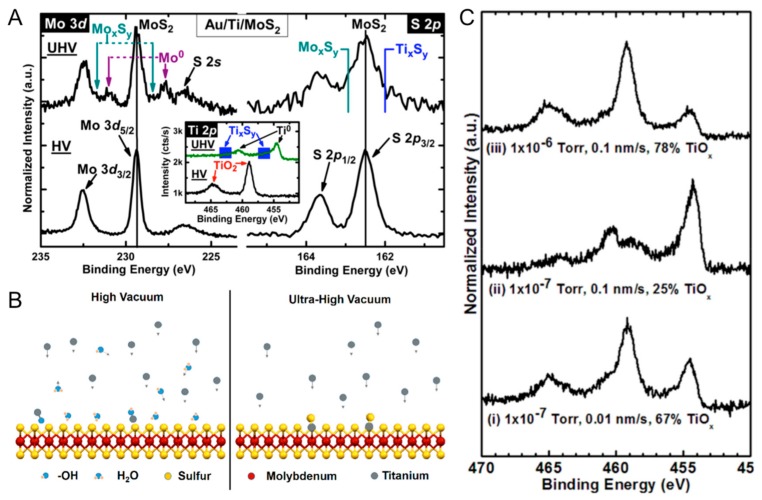
(**A**) Mo 3d, S 2p, and Ti 2p (inset) for UHV Ti–MoS_2_ exposed to air for 20 minutes. The new high binding energy features in the Ti 2p spectra can be attributed to partial oxidation of some of the titanium species. However, the presence of Ti_x_S_y_ is still clearly detected in all three core-levels. (**B**) schematic of depositions in high vacuum (HV) with oxidizing species present versus ultra-high vacuum (UHV) with no such species present. (**C**) Ti 2p core-level spectra for Ti deposited onto samples cut from a single Gr–SiO_2_ sample at different deposition conditions resulting in different oxide compositions. Parts A and B Reprinted and adapted with permission from McDonnell et al. *ACS Applied Materials & Interfaces* **8**, 8289–8294 (2016). Copyright (2016) American Chemical Society. Part C Reprinted and adapted with permission from Freedy et al. *Nanotechnology* **29**, 145201 (2018). Copyright (2018) IOP Publishing Ltd.

As mentioned earlier, the only contradiction between the work of McGovern et al. [16] and Lince et al. [28] was that McGovern observed expected reactions between Ti and MoS_2_ but Lince et al. did not. Lince et al. attributed this to the potential oxidation of Ti by the deposition in a poorer vacuum environment. More recent investigations published by McDonnell et al. [18]. and Smyth et al. [24,67,75]. demonstrate that the chamber pressure during contact deposition, a process parameter that is typically unreported in device papers, has a measurable impact on the chemistry of the interface. In addition to affecting the concentration of carbon in the metal film or at the interface, the base pressure determines which chemical states will be present at the metal-TMDC interface. In the case of Ti, for example, the presence of oxidizing species in HV deposition chambers prevents chemical reactions between Ti and MoS_2_ as Ti instead reacts with these molecules to form TiO_2_ [18]. This is illustrated in Figure 4a. The author noted, that when Ti was deposited in HV, the MoS_2_ did not exhibit changes in its chemical state. However, in UHV, the expected formation of metallic Mo and titanium sulfides was found in agreement with McGovern et al. [16]. The authors proposed that under HV conditions, there is a sufficient partial pressure of oxidizing species present that they will be impinge on the surface of MoS_2_ at rates comparable to a monolayer per second. With Ti deposition rates on the same order of magnitude, this essentially amounts to a co-deposition of Ti and Oxygen when the deposition is carried out in HV. The differences between HV and UHV depositions are illustrated schematically in Figure 4b. XPS was used to verify that Ti deposition in HV can be completed oxidized (inset of Figure 4a). More recent work by Freedy et al. [73] further tested this hypothesis by examining the Ti chemistry as a function of vacuum pressure and deposition rate in HV. These result (shown in Figure 4c) showed that the Ti metal to Ti oxide ratio could be readily altered by varying either vacuum pressure or Ti deposition rate, which is consistent with the model proposed by McDonnell et al. [18].

### 5.2. Engineering the Interface

One method for controlling interface chemistry is to decouple the metal contact from the TMDC via an interfacial oxide layer. Improvements in electrical contact resistance, device stability, on-current, and mobility via this method using Ti-TiO_2_ contacts have been demonstrated in a number of publications [76,77,78,79]. The success of the interfacial oxide approach has been attributed to Fermi level de-pinning by Park et al. [76] and Kim et al. [78] by electrical measurements of the Schottky barrier height. The presence of an oxide is said to block the penetration of the metal wave function into the semiconductor, preventing metal-induced gap states which pin the Fermi level. Another possible mechanism discussed by Kaushik et al. [79] is the lowering of the electron Schottky barrier due to n-type charge-transfer doping from the oxide to MoS_2_. The effects of interfacial oxide on interface chemistry and transport properties was recently expanded on by Freedy et al. [80].

By employing in-situ UHV Ti deposition and characterization of MoS_2_, Freedy et al. [80] were able to use partial pressures of O_2_ during deposition in order to protect the MoS_2_. The resultant TiO_x_ thin films were deposited by deliberate reactive e-beam of Ti rather than the accidental reactive e-beam that takes place in HV reactors. The authors showed that even a thin 1 nm layer of TiO_x_ was sufficient to protect the MoS_2_ interface from reactions, and that subsequent Ti deposition could be carried out with no oxygen to ensure that the remainder and topmost portion of the contact is metallic Ti. By using the ex-situ thermal characterization techniques of time dependent thermoreflectance (TDTR), the authors were able to show how important such a Ti-TiO_x_-MoS_2_ structure may be for Ti contact adhesion layers.

**Figure 5:**
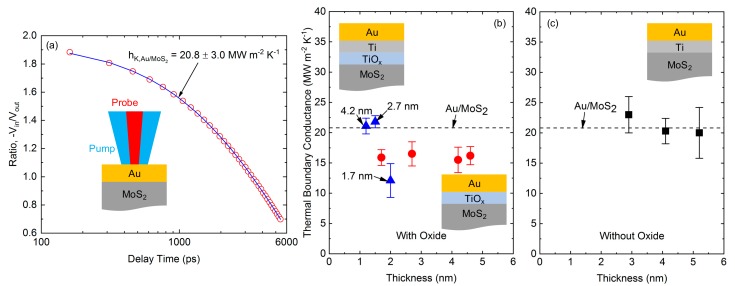
(**a**) TDTR data and best fit for the Au–MoS_2_ structure. Thermal boundary conductance as a function of interfacial layer thickness for the MoS_2_ substrates (**b**) with and (**c**) without an oxide interlayer. Samples included are Au–Ti (black squares), Au–TiO_x_ (red circles), and Au–Ti–TiO_x_ (blue triangles) in addition to a reference sample of Au–MoS_2_ (dashed line). The arrows indicate the Ti metal thickness for each Ti–TiO_x_ sample where data are plotted as a function of oxide thickness. Reprinted with permission from Freedy et al., Phys Rev Materials, 3, 104001 (2019), DOI: 10.1103/PhysRevMaterials.3.104001 Copyright (2019) by the American Physical Society.

Like contact resistance, thermal boundary conductance is an important property for nanoelectronic devices. This is because heat dissipation is a major issue in transistors and low boundary conductances can lead to localized heating of the devices, compromising performance and reliability. In a typical Au-Ti-MoS_2_ stack, it is now known that deposition of contacts in UHV will lead to Ti-MoS_2_ reactions that limit device performance [18,19]. In the recent work of Freedy et al. [80] it was shown that, while the Au-TiO_x_-MoS_2_ structure prevented reactions at the Ti-MoS_2_ interface, the thermal boundary conductance was markedly lower than that of the Au-Ti-MoS_2_ structure. This is shown in Figure 5. However, utilizing a Au-Ti-TiO_x_-MoS_2_ structure yielded a protected MoS_2_ interface while providing comparable thermal boundary conductance to the Au-Ti-MoS_2_ structure. This work showed that the metal-adhesion layer interface can be critical to heat dissipation and should not be overlooked. Furthermore, the use of a metal-oxide heterostructure (Ti-TiO_x_) adhesion preserved both the semiconductor chemistry and the thermal transport properties of the contact, offering a practical engineering solution for MoS_2_ contacts.

### 5.3. Thermal Stability

Annealing the device after contact deposition is common practice in device processing and notable changes in device transport properties after annealing have been observed. [19,35,75,81,82] English et al. report that post-deposition annealing reduces hysteresis and stabilizes electrical measurements for Au contacted FETs [19]. Baugher et al. claim that vacuum annealing of devices with Ti-Au contacts eliminated all Schottky behavior [82]. Abraham and Mohney observe decreased contact resistance by rapid thermal annealing of MoS_2_ FETs with Ag contacts [35]. The improvement is speculated to be due to the diffusion of Ag into MoS_2_, resulting in local doping under the contact, which would be consistent with prior reports from Li et al. [38] of Ag diffusion into MoS_2_ at temperatures >326 °C. Liu et al. [83] show a current improvement of two orders of magnitude after vacuum annealing WS_2_ FETs with Ti-Au contacts, stating that annealing enhances contact adhesion. In all of the examples mentioned, chemical characterization of the interface is lacking while the observed improvements are almost certainly correlated with changes in interface chemistry. Recently, Smyth et al. [75] reported substantial improvement after annealing WSe_2_ FETs contacted with Pd. They find that annealing Pd-WSe_2_ in forming gas at 400 °C drives the formation of PdSe_x_ which results in Ohmic band alignment. They also note that annealing in UHV results in a smaller composition of PdSe_x_ and a higher Schottky barrier in comparison with annealing in forming gas. It is clear that post-deposition annealing conditions also play an important role in determining contact properties.

In the previously described study of Cr on MoS_2_ by Durbin et al. [26], it was shown that Cr is reactive with MoS_2_ at room temperature forming metallic Mo and Cr-S, and that heating the material following deposition resulted in an increase in the reaction products. By varying the incident photon energy, they acquired a non-destructive depth profile and concluded that the resultant structure consisted of MoS_2_ covered with a clustered or islanded Cr-Mo alloy, covered with a Cr-S film that possibly contained Cr metal, and was terminated with a sulfur rich Cr-S surface. Lince et al. [29,30] report similar behavior for Mn on MoS_2_, with the reaction driven to completion by 497 °C followed by Mn agglomeration at 767 °C. Unlike Mn, Fe was found to delaminate from the MoS_2_ surface as a result of annealing. These studies illustrate the differences in the behavior of reactive (Cr and Mn) vs. non-reactive (Fe) metals on MoS_2_ after thermal annealing. As mentioned in relation to the device studies of Ag-MoS_2_ contacts by Abraham and Mohney [35] the prior work by Li et al. [38] had shown that Ag diffused into MoS_2_ at temperature >326 °C and that there was an associated charge transfer from Ag to MoS_2_ detected by XPS binding energy shifts. Notably, this shift reversed itself after annealing to 526 °C and Ag clusters formed after annealing to 577 °C.

**Figure 6:**
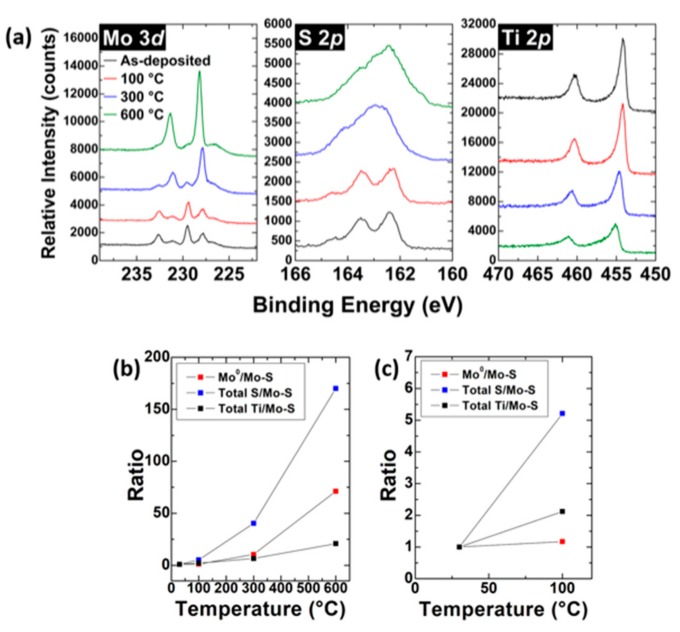
(**a**) XPS spectra acquired following 30 min anneals at each temperature. These were performed sequentially on the same sample. (**b**) Intensity ratios based on the data in (**a**) where (**c**) highlights the changes that occur at 100 °C. Reprinted with permission from Freedy et al. ACS Appl. Mater. Inter. 11(38) 35389, (2019). Copyright (2019) American Chemical Society.

In recent work by Freedy et al. [69,84] the stability of the metal-MoS_2_ interface has been considered. In particular, the Ti-MoS_2_ interface which forms in UHV was studied as a function of annealing temperature. The authors found that detectable concentration of metallic Mo and TiS_x_ species increased after anneals as low as 100 °C (the lowest temperature employed in the study) which is reproduced in Figure 6. This would suggest that such contact would not be stable during back-end process and may in fact be unstable during some operating conditions. This result may explain why Radisavljevic reports that Au-Ti contact performed better before annealing [14].

**Figure 7:**
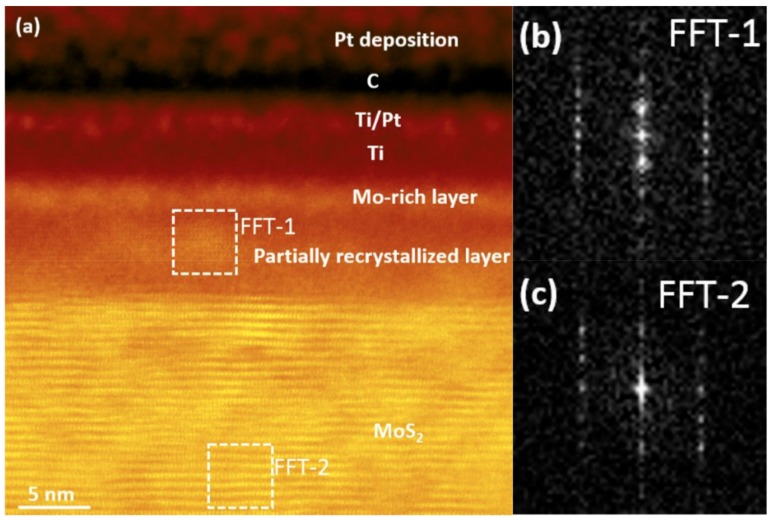
(**a**) Cross-sectional ADF-STEM image of Ti–MoS_2_ after 30 min anneal at 400 °C showing a Mo-rich layer and a partially recrystallized layer grown out from the disordered Mo/S-rich layer, (**b**) and (**c**) are FFT images of the white dotted-line framed regions in (a). Reprinted with permission from Freedy et al. ACS Appl. Mater. Inter. 11(38) 35389, (2019). Copyright (2019) American Chemical Society.

This work also investigated the structure and composition profile across the interface before and after annealing at 400 °C by high-resolution transmission electronic microscopy (HRTEM). The initial result was similar to that reported by Wu et al. [17] and showed that the Ti diffuses into the MoS_2_. However, additional information about the Ti metal region was gathered and it was shown that the Mo and S also diffuse outward into the Ti layer. The HRTEM acquired after annealing are reproduced in Figure 7. Following annealing, there is a clear Mo rich region that separated the region of MoS_2_ (with Ti impurities) and Ti (with Mo and S impurities). Also observed is clear evidence of recrystallization in the MoS_2_ region that was disturbed by Ti diffusion. Whether this is MoS_2_, TiS_2_ or MoTiS_2_ cannot be conclusively determined at this time.

## 6. Conclusions

A substantial volume of recent work in the literature is focused on the synthesis and characterization of 2D materials and on the fabrication and characterization of devices using 2D materials. Some studies have focused on optimizing transport properties of 2D-contact interface while a very small number of recent papers have specifically examined the chemistry of the interface. There exists a gap between these two topics of research, resulting in a lack of understanding of the relationship between contact processing, interface chemistry, and electrical and thermal transport properties. While the reactive nature of the metal-2D interface has been previously documented, the chemical composition of the interface and effects of processing has only very recently been explored in greater detail for the Ti-MoS_2_ interface. Recent work discussed in this review has demonstrated that various aspects of processing, such as deposition conditions and post-deposition annealing, can have drastic effects on interface chemistry and transport properties. This provides a more complete approach to the interpretation of the behavior of electronic devices, particularly when discrepancies are observed between theory and experiment or between experiments as summarized in Table 2. Furthermore, control over interface chemistry during processing opens doors for interface engineering, which can be implemented to tailor thermal and electrical transport across interfaces to meet device-specific requirements and expand the field of 2D nanoelectronics into new domains.

## Figures and Tables

**Table 1:** Summary of literature on experimental chemical and electronic characterization of metal–MoS_2_ interfaces.

	Ref.	Deposition	Annealing	Characterization	Key Result
**Ti**	[16]	UHV	None	XPS	Reaction of Ti+MoS_2_ →Ti–S + Mo^0^ at room temperature
	[17]	UHV	None	TEM, EELS	Reaction of Ti+MoS_2_ →Ti–S + Mo^0^ at room temperature
	[18]	HV and UHV	None	XPS	Reaction occurs in UHV only and not in HV deposition
	[19]	UHV	In total, 300 °C for 2 h in HV	TLM	High RC (~7–9 kΩ µm)
	[20]	Unreported	None	FET I-V Curves	EF pinned near MoS_2_ conduction band (Φ=0.050 eV)
**Ni**	[21,22] [22]	UHV	Heated sequentially in UHV to 927 °C; time not specified	Auger electron spectroscopy (AES)	- No interactions below 327 °C - Some diffusion of Ni into MoS_2_ at 327–527 °C - Agglomeration of Ni film > 527 °C
	[19]	HV	In total, 300 °C for 2 h in HV	TLM	RC ~ 4–7 kΩ µm
	[20]	Unreported	None	FET I-V Curves	n-type Fermi-level pinning (Φ = 0.150 eV)
**Au**	[23]	UHV	None	XPS	No chemical bonding
	[24]	HV and UHV	None	XPS	No chemical bonding
	[19]	HV and UHV	In total, 300 °C for 2 h in HV	TLM FET- IV curves	- RC for Au ~0.7–2 kΩ µm in UHV; ~3.5–5 kΩ µm in HV - Φ = 0.15 eV
	[25]	Unreported	Unreported	TLM FET I-V Curves	- RC ~ 30–45 Ω mm - Φ = 0.12 eV
**Cr**	[26,27]	UHV	Heated sequentially in UHV from 425–850 °C, Time not specified	XPS	- Reaction of Cr+MoS_2_ →Cr-S + Mo^0^ at room temperature - Reaction driven to completion < 425 °C - Increase in S composition at the Cr surface with temp. - Coalescence of Cr > 650 °C
	[24]	HV and UHV	None	XPS	- Reaction occurs under both HV and UHV conditions - Both depositions result in Mo^0^ and Cr_x_S_y_ - HV deposition conductions also result in Cr_x_O_y_
**Mn**	[28]	HV	None	XPS	Chemical reaction observed
	[29]	HV and UHV	Heated sequentially in UHV from 497 to 857 °C, time not specified	XPS	- Reaction of Mn+MoS_2_ →Mn-S + Mo^0^ as deposited - Reaction driven to completion above 497 °C - Increase in S composition at the Mn surface with temp. - Coalescence of Mn > 767 °C
**Fe**	[30]	UHV	Heated sequentially in UHV from 327 to 927 °C, time not specified	XPS	- No evidence of reaction in the bulk - Fe-S surface states and S-vacancy states are observed following initial deposition - Heating eliminates these chemical states
	[31]	UHV	UHV at 927 °C for a few minutes; repeated 20 times	AES with Ar^+^ depth profiling	- Intercalation of Fe between MoS_2_ layers due to annealing - Potential formation of FeMo_2_S_4_
**Pd**	[28]	HV	None	XPS	No chemical bonding
	[23]	UHV	None	XPS	No chemical bonding
	[32]	UHV	None	XPS	- No chemical bonding - Perturbation of the MoS_2_ surface due to Pd overlayer - Pd aligns midgap with MoS_2_ (Φ = 0.67 eV)
	[25]	Unreported	Unreported	TLM FET I-V Curves	- RC ~ 75–200 kΩ mm - Φ = 0.4 eV
	[31]	UHV	UHV at 927 °C for a few minutes; repeated 20 times	AES with Ar^+^ depth profiling	-Diffusion of Pd into MoS_2_ layers due to annealing; uniformly distributed in the bulk unlike Fe
**Al**	[28]	HV	None	XPS	No chemical bonding
	[16]	UHV	None	XPS	No chemical bonding
	[33]	Unreported	In total, 110 °C for 15 h in HV	FET I-V Curves	Significant electron doping manifested in no OFF state
**In**	[28]	HV	None	XPS	No chemical bonding
**Mg**	[16]	UHV	None	XPS	Evidence of chemical bonding
**Mo**	[34]	Unreported	In total, 146 °C for 2 h	FET I-V Curves	- RC ~ 2 kΩ μm
**Ag**	[35]	HV	In total, 150 °C for 24 h in HV followed by RTA in Ar at 200–500 °C	TLM FET I-V Curves	- Negligible reduction in RC after 24 h HV anneal at 150 °C - RC reduced from ~2 kΩ μm to 0.2–0.7 kΩ μm after annealing in RTA at 200–500 °C - Reduction in RC is attributed to diffusion of Ag resulting in doping
	[36]	Unreported	None	FET I-V Curves	- 60x larger ON current than Ti contacted devices
	[37]	Unreported	In total, 400–600 °C for 5 min	Radioactive tracer	Diffusion of Ag into MoS_2_ crystal results in intercalation between layers; no diffusion detected in-plane
	[38]	UHV	In total, −173 to 577 °C in UHV, time not specified	XPS and AES	- No reaction from −173 to 27 °C - Diffusion of Ag into bulk after annealing to 326 °C; negative binding energy shift due to silver→sulfide charge transfer - Heating above 526 °C restored binding energies to pre-anneal positions likely due to the diffusion of Ag into MoS_x_ or the formation of AgMoS_x_. - Ag clusters form on surface after annealing to 577 °C
**Sc**	[20]	Unreported	None	FET I-V Curves	EF pinned near MoS_2_ conduction band (Φ = 0.030 eV)
	[24]	HV and UHV	None	XPS	- Reaction occurs under both HV and UHV conditions - HV deposition results in MoO_x_, MoO_x_S_y_, and Sc_x_O_y_ - UHV deposition results in Mo^0^ and ScS_x_ and
**Pt**	[20]	Unreported	None	FET I-V Curves	n-type Fermi-level pinning (Φ = 0.230 eV)
**Ir**	[24]	HV and UHV	None	XPS	- Reaction occurs under both HV and UHV conditions - Both depositions result in MoS_x_ and IrS_x_ - HV deposition also results in IrO_x_

**Table 2:** Summary of property variability reported and possible explanations.

	Ref	Property	Ref	Property	Suggested Explanation
**Metal induced doping in MoS_2_**	[25]	n-type Pd-MoS_2_ interface	[64]	p-type Pd-MoS_2_ interface	MoS_2_ variability, since Au on MoS_2_ can exhibit both n-type and p-type contact behavior. [23]
**Trends in metal-MoS_2_ Schottky barriers and contact resistance**	[20]	Schottky barrier for Ti-MoS_2_ lower than for Ni-MoS_2_	[19]	Contact resistance for Ni-MoS_2_ lower than for Ti-MoS_2_	Ti used by Das et al. [20] was likely oxidized during high vacuum deposition. Ti used by English et al. [19] likely resulted in Ti-MoS_2_ interactions during ultra-high vacuum deposition [18]
**Fermi-level pinning at the metal-MoS_2_ interface**	[23]	Metal-MoS_2_ interface is unpinned based on photoemission	[20]	Devices clearly behave as if the Fermi-level is pinned	Not current resolved. However, parallel conduction paths suggested previously [23] could explain why contacts would appear pinned in device, but would not appear pinned by photoemission.
